# Inferring gene regulatory networks from single-cell transcriptomics based on graph embedding

**DOI:** 10.1093/bioinformatics/btae291

**Published:** 2024-05-29

**Authors:** Yanglan Gan, Jiacheng Yu, Guangwei Xu, Cairong Yan, Guobing Zou

**Affiliations:** School of Computer Science and Technology, Donghua University, Shanghai 201620, China; School of Computer Science and Technology, Donghua University, Shanghai 201620, China; School of Computer Science and Technology, Donghua University, Shanghai 201620, China; School of Computer Science and Technology, Donghua University, Shanghai 201620, China; School of Computer Engineering and Science, Shanghai University, Shanghai 200444, China

## Abstract

**Motivation:**

Gene regulatory networks (GRNs) encode gene regulation in living organisms, and have become a critical tool to understand complex biological processes. However, due to the dynamic and complex nature of gene regulation, inferring GRNs from scRNA-seq data is still a challenging task. Existing computational methods usually focus on the close connections between genes, and ignore the global structure and distal regulatory relationships.

**Results:**

In this study, we develop a supervised deep learning framework, IGEGRNS, to infer GRNs from scRNA-seq data based on graph embedding. In the framework, contextual information of genes is captured by GraphSAGE, which aggregates gene features and neighborhood structures to generate low-dimensional embedding for genes. Then, the *k* most influential nodes in the whole graph are filtered through Top-k pooling. Finally, potential regulatory relationships between genes are predicted by stacking CNNs. Compared with nine competing supervised and unsupervised methods, our method achieves better performance on six time-series scRNA-seq datasets.

**Availability and implementation:**

Our method IGEGRNS is implemented in Python using the Pytorch machine learning library, and it is freely available at https://github.com/DHUDBlab/IGEGRNS.

## 1 Introduction

The identity and behavior dynamics of cells are governed by complex gene interactions, which in turn define cellular morphology and functions (Daskalaki *et al.* 2018). Gene regulatory networks (GRNs) can model the causal regulatory relationships between transcription factors (TFs) and their target genes. In GRNs, the regulatory relations among genes are represented as graphs, where nodes are regulators and their target genes, and edges represent that there exist regulatory relationships between genes. They have become essential tools for interpreting biological processes and identifying molecular regulators and biomarkers in complex diseases (Seçilmiş *et al.* 2022). Therefore, inferring GRNs based on gene expression profiles is a long-standing computational challenge in system biology research field (Iacono *et al.* 2019).

A plethora of computational approaches have been developed for inferring GRNs from bulk expression data and single-cell RNA-seq data (Pratapa *et al.* 2020, Zhao *et al.* 2021). Generally, the existing methods can be classified into two main categories, unsupervised methods and supervised methods (Bravo González-Blas *et al.* 2023). Unsupervised methods explore underlying patterns and structures from the gene expression data, and then infer regulatory interactions without a known network. GENIE3 is based on decision trees, which infer the regulatory interactions of each gene independently by an integrated tree-based approach (Huynh-Thu *et al.* 2010). PPCOR infers GRNs by calculating partial and semipartial correlation coefficients between genes (Kim 2015). PIDC utilizes the multivariate information theory to reconstruct undirected regulatory networks among genes (Chan *et al.* 2017). SCODE infers regulatory networks based on ordinary differential equations and linear regression (Matsumoto *et al.* 2017). SINCERITIES quantifies the distance between two cumulative distribution functions of gene expressions from subsequent time points, and employs regularized linear regression to infer directed regulatory relationships among genes (Papili Gao *et al.* 2018). BiXGBoost respectively infers the regulatory and regulated relationships of genes through gradient boosting decision trees, and integrates the forward and reverse relationships to generate consistent gene score ranking relationships.(Zheng *et al.* 2019). DeepSEM is also an unsupervised method based on beta-variant self-encoder, whose encoder takes the expression profile of one gene at a time as the input feature of the neural network, and later learns gene interaction relationships through a multilayer perceptron (Shu *et al.* 2021). Although much progress has been made, inferring GRNs from scRNA-seq data is still challenging, due to its high sparsity, noise, and dropout events.

Different from unsupervised methods, supervised methods exploit on not only gene expression profiles, but also prior information to infer GRNs, such as known gene interactions, organism, or tissue information (Razaghi-Moghadam and Nikoloski 2020). Recently, as deep learning models can better handle large-scale and high-dimensional data, researchers gradually rely on the powerful representation learning capability of deep neural networks to capture complex nonlinear relationships and infer GRNs from gene expression profiles (Muzio *et al.* 2021, Greener *et al.* 2022). As an early supervised algorithm, CNNC transforms the coexpression data of gene pairs into histograms, and then deep convolutional neural networks (CNNs) are utilized to learn the relationships between genes. However, it is time-consuming to transform numerous gene pairs into matrices (Yuan and Bar-Joseph 2019). GNE uses gene expression profiles and network topology to predict gene interactions through ANN (Kc *et al.* 2019). TDL devises a supervised framework which represents the data as 3D tensors and trains convolutional and recurrent neural networks for predicting interactions (Yuan 2021). DGRNs are a hybrid deep learning model that effectively extracts temporal information, which constructs an input expression matrix by extracting special correlation vectors representing gene expression features, and inferring temporal and spatial features through GRU and CNN, respectively (Zhao *et al.* 2022). DeepRIG first constructs a prior regulatory graph by transforming the gene expression profiles into the coexpression mode, then adopts a graph autoencoder model to learn gene latent embeddings and to infer the GRN (Wang *et al.* 2023). STGRNs is a transformer-based method for inferring GRNs from scRNA-seq data. It converts gene pairs into contiguous subvectors, which can be used as input for the transformer encoder (Xu *et al.* 2023). In general, these deep learning based methods infer GRNs through two primary steps, first converting gene expression data into a suitable data format, and then employing deep neural network models to predict the regulatory relationships. Although these deep neural networks have achieved notable success in various biological tasks, these CNN model-based methods still encounter with some limitations in GRN inference. On one hand, the generation of image data not only gives rise to unanticipated noise but also conceals certain original data features. On the other hand, since this procedure alters the format of the data, the results predicted by these approaches is lack of biological explainability.

To address these limitations, we propose a supervised deep learning framework, IGEGRNS, to infer GRNs from scRNA-seq data through graph embedding. We convert GRN inference task to linkage prediction problem, predicting the existence of a directed edge between TFs and target genes. IGEGRNS formulates gene–gene relationships with graph neural networks, and learns low-dimensional embeddings of gene pairs using GraphSAGE. Contextual information of genes is captured by GraphSAGE, which aggregates gene features and neighborhood structures to generate low-dimensional embedding for genes. Meanwhile, the *k* most influential nodes in the whole graph are filtered through Top-k pooling. Then, the regulatory relationships between TFs and target genes are further learned by stacking CNNs, which enhance the network representation and better adapt to complex input data by extracting features layer by layer. Compared with nine competing supervised and unsupervised methods, our approach achieves better performance on six time-series scRNA-seq datasets.

## 2 Materials and methods

### 2.1 The IGEGRNS framework

**Figure 1.**
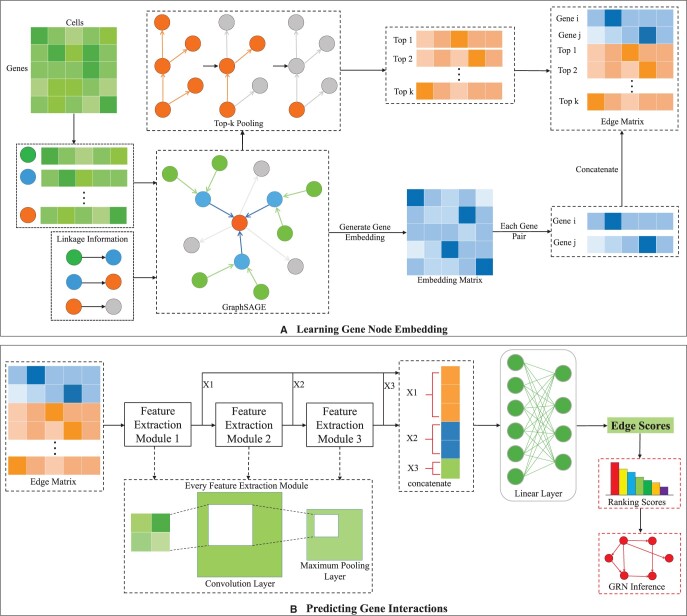
The overview of IGEGRNS. IGEGRNS is composed of two main modules: (A) Learning gene node embedding module. To learn the low-dimensional embedding of genes, GraphSAGE is adopted to iteratively aggregate the node feature and its l-hop neighboring nodes. Meanwhile, Top-k pooling filters the top *k* nodes with the highest influence on the whole graph. (B) Predicting gene interactions module. This module consists of stacked CNNs and a fully connected layer. Stacked CNN further learns the high-level representation of the gene pairs, which is used to predict the regulatory relationship between TFs and target genes

As predicting the regulatory relationships among genes is essential for inferring GRNs from observed gene expression data, linkage prediction is a fundamental problem in the study of GRNs. IGEGRNS converts the GRNs inference into a linkage prediction problem, determining whether there are regulatory edges between TFs and target genes. As illustrated in Fig. 1, IGEGRNS is a supervised deep learning framework, inferring GRNs from scRNA-seq data through graph embedding. Overall, the linkage prediction process can be divided into two main steps. First, the embedding of gene pairs is learned through GraphSAGE (Hamilton *et al.* 2017). Based on the gene expression data and prior knowledge, GraphSAGE generates low-dimensional embedding for genes, iteratively aggregating the information of gene nodes and their neighboring nodes. Meanwhile, Top-k pooling filters the top *k* nodes with the highest influence on the whole graph (Gao and Ji 2019). Then we concatenate the corresponding embedded vectors of the gene pairs and the feature vectors of the selected top *k* gene nodes. Second, based on the concatenated feature matrix, we predict whether there is a regulatory edge between each gene pair. The prediction module consists of a stacked 3-layer CNN and a fully connected layer. For the stacked 3-layer CNN, each feature extraction layer includes a regularization layer, a CNN layer, and a maximum pooling layer. Further, the high-level features learned from these three feature extraction layers are concatenated and fed into the fully connected layer, and scored by the Sigmoid function.

#### 2.1.1 Learning gene node embedding

The original gene expression data is a matrix of n×m, where *n* denotes the number of genes and *m* refers to the number of cells. Our task is to reconstruct GRNs based on the given gene expression profiles. To learn the low-dimensional embedding of gene nodes, we adopt GraphSAGE to perform graph embedding. Due to its versatility in neighbor sampling and aggregation techniques, GraphSAGE enables effective learning of both local and global features within the network topology (Hamilton *et al.* 2017). By leveraging GraphSAGE, the proposed model can better capture the intricate interactions between genes, resulting in high-quality embedding vectors derived from gene expression data and network interactions.

For each node v(v∈{1,2,3,…,n}), N(v) is referred to the neighbor set of node *v*. Here, the mean function is chosen as the aggregation function, and then the d-dimensional neighbor node aggregation of node *v* is represented as:

(1) $$h_{N(v)}^{l}=\frac{1}{\left|N(v)\right|}\sum_{u\in N(v)}h_{u}^{l},$$

where *l* represents the number of hops of neighboring nodes that each vertex can aggregate.

The embedding of node *v* is learned based on its *l*-hop neighboring nodes. Therefore, a new vector $h_{v}^{l}$ can be used to represent the embedding of node *v*, which captures the *l*-hop neighborhood information of node *v*:

(2) $$h_{v}^{l}\leftarrow \sigma \left(W\cdot \mathrm{CONCAT}\left(h_{v}^{l-1},h_{N(v)}^{l}\right)\right),$$

where σ denotes a Relu activation function, *W* denotes the parameter matrix to be learned, and *CONCAT* represents the concatenating operation.

Based on the given gene expression matrix and the prior link knowledge, GraphSAGE conducts graph embedding and learns a low-dimensional embedded representation for each gene node. By leveraging the known regulatory relationships in the training set, the model can effectively learn accurate embedding vectors for gene nodes, consequently facilitating the inference of GRNs. Then, the embedding matrix is composed of all gene node embeddings is represented as $X=\left\{h_{1}^{l},h_{2}^{l},\ldots ,h_{n}^{l}\right\}$, $X\in R^{n\times d}$, where *d* denotes the feature dimension of the gene node.

**Figure 2.**
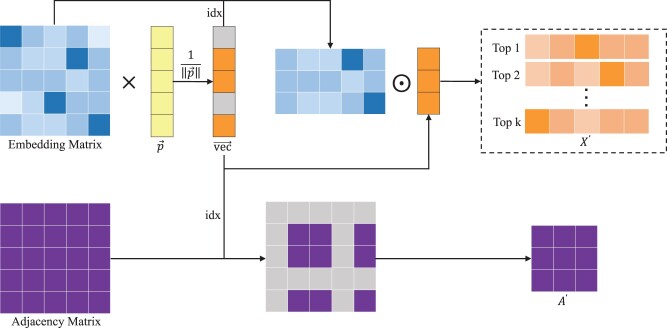
Illustration of Top-k pooling process. Top-k pooling filters the *k* most influential nodes based on the embedding matrix generated by GraphSAGE

Meanwhile, We adopt Top-k pooling strategy to select the top *k* nodes with the highest influence on the whole graph (Gao and Ji 2019). As illustrated in Fig. 2, the detailed process is formulated as below:

(3) $$\vec{\mathrm{vec}}=\sigma \left(\frac{X\vec{p}}{\left\|\vec{p}\right\|}\right),$$

(4) $$\vec{\mathrm{idx}}=\mathrm{topk}\left(\vec{\mathrm{vec}}\right),$$

(5) $$X^{\prime }={\left(X⊙\tanh\left(\vec{\mathrm{vec}}\right)\right)}_{\vec{\mathrm{idx}}},$$

(6) $$A^{\prime }=A_{\vec{\mathrm{idx}},\vec{\mathrm{idx}}},$$

where σ denotes a Relu activation function, where $\vec{p}$ is the learnable vector, and the projection fraction of *p* is used to determine which node to be discarded. ‖·‖ denotes the L2-norm operator, *topk* function selects the top-k index from the input vector, $\vec{\mathrm{idx}}$ is an index operation to obtain slices according to the specified index, and ⊙ is the element multiplication.

Next, we construct the input matrix for the subproblem of determining the existence of a directed edge from gene *i* to gene *j*. The input matrix $E_{i,j}$ consists of k+2 vectors, including the embedding vector $h_{i}^{l}$ of gene *i*, $h_{j}^{l}$ of gene *j*, and the vectors $h_{t_{1}},h_{t_{2}},\ldots ,h_{t_{k}}$ obtained by Top-k pooling, which is represented as:

(7) $$\begin{array}{c} E_{i,j}=\left[\begin{array}{c} h_{i}^{l}\\ h_{j}^{l}\\ h_{t_{1}}\\ \ldots \\ h_{t_{k}} \end{array}\right], \end{array}$$

where i,j∈v, $E_{ij}$ is a (k+2) × d matrix.

#### 2.1.2 Predicting gene interactions

The core of the gene linkage prediction module consists of a stacked three-layer CNN and a fully connected layer. Specifically, the stacked CNN is introduced to conduct further feature extraction, which is a three-layer CNN stacking model. Each feature extraction layer includes a regularized BatchNorm layer, a CNN convolutional layer and a maximum pooling layer. The outputs of these three feature extraction layers are concatenated before feeding into the fully connected layer. The purpose of this step is to further learn the high-level feature representation, increase the nonlinear capability of the network, and better capture the complex relationships hidden in the data. The concatenated vectors are fed into the lazy linear layer and the ordinary linear layer, and finally the edges $e_{ij}$ are scored by the Sigmoid function. The predicted scores, denoted as $\hat{y_{i}j}$, are normalized in the interval [0,1], which represents the probability that TF *i* regulates target gene *j*. We train the model using a binary cross-entropy loss function as below:

(8) $$\begin{array}{c} \begin{array}{c} \text{BCELoss}=-\frac{1}{N}\sum_{s=1}^{N}y_{s}\cdot \;\text{log}\;\left(\hat{y_{s}}\right)\\ +\left(1-y_{s}\right)\cdot \;\text{log}\;\left(1-\hat{y_{s}}\right). \end{array} \end{array}$$

where *N* denotes the number of samples involved in training, $y_{s}$ denotes the true label of the *s*th sample, and $\hat{y_{s}}$ denotes the model prediction.

### 2.2 Datasets

The proposed method IGEGARNS is evaluated on six time-series scRNA-seq datasets, including human embryonic stem cells (hESCs), human mature hepatocytes (hHEP), mouse embryonic stem cells (mESC), mouse hematopoietic stem cells with an erythroid-lineage profile (mHSC-E), mouse hematopoietic stem cells with a granulocyte-monocyte profile (mHSC-GM) and mouse hematopoietic stem cells with a lymphoid lineage (mHSC-L). For these datasets, the corresponding cell-type-specific networks provided in previous studies are regarded as the reference network for the evaluation (Xu *et al.* 2013, Oki *et al.* 2018, Moore *et al.* 2020). We preprocess these six scRNA-seq datasets and infer the gene interactions as BEELINE (Pratapa *et al.* 2020). We respectively select 500 and 1000 the most differential genes for the inference of GRNs.

For the proposed method, the inference of GRNs is transformed into a linkage prediction problem, predicting whether there are directed regulatory edges for gene pairs. Therefore, we evaluate the supervised method by 5-fold cross-validation. We define the TFs appearing in the reference network as TFs and the target genes as targets. We consider the edges existing in the reference network as positive. Otherwise, the edges are regarded as negative. Generating negative edges can help the algorithm to better distinguish the real regulatory relationships, and reduce the sensitivity to random noise, and thus optimize the algorithm performance. We mix positive and negative edges and randomly divide them into five equal parts. We choose four parts as the training set and the remaining part as the test set. We average the results of 5-fold cross-validation to obtain the final AUROC and AUPRC scores. For the compared unsupervised model, we use the results of the framework BEELINE (Pratapa *et al.* 2020).

### 2.3 Performance metrics

To compare the performance of different methods in inferring GRNs, we adopt two commonly used metrics AUROC and AUPRC. For GRN inference, changing the thresholds leads to different GRNs, and AUROC and AUPRC allow comparing the performance of different GRN inference algorithms under different thresholds. Specifically, ROC is a curve with false positive rate as the horizontal axis and true positive rate as the vertical axis. AUROC is the area under the ROC curve, and AUPRC is the area under the Precision-Recall curve. Higher AUROC and AUPRC scores indicate better performance.

(9) $$\text{FPR}=\frac{\text{FP}}{\text{FP}+\text{TN}},$$

(10) $$\text{TPR}=\frac{\text{TP}}{\text{TP}+\text{FN}},$$

(11) $$\text{Precision}=\frac{\text{TP}}{\text{TP}+\text{FP}},$$

(12) $$\text{Recall}=\frac{\text{TP}}{\text{TP}+\text{FN}}.$$

where TP denotes the number of true regulatory edges that are predicted to be positive, FN denotes the number of true regulatory edges that are predicted to be negative, FP denotes the number of false regulatory edges that are predicted to be positive, and TN denotes the number of false regulatory edges that are predicted to be negative.

In our experiments, we average the results of 5-fold cross-validation to obtain the final AUROC and AUPRC scores.

## 3 Results

### 3.1 Implementation details

The proposed method adopts GraphSAGE to aggregate gene features and neighboring nodes, and to generate low-dimensional embedding. Specifically, the output dimension is set to 256, the aggregator function is MEAN aggregator function. We aggregate 1-hop neighbor node information. To evaluate the efficacy of different neighbor node configurations, we conduct experiments utilizing 1-hop, 2-hop, and 3-hop neighbor nodes. The results demonstrate that aggregating 1-hop neighbors achieves better performance, indicating that the representation of nodes can be well represented by the information of their direct neighbors. Aggregating neighbors at a further distance may increase computational costs and introduce sparsity during information propagation. For the Top-k pooling strategy, the parameter *k* is set to 1. The size of the CNN convolution kernel is set to 2. In addition, our learning rate is initially set to 0.01, and with every 10 epochs of training, the learning rate decreasing to 80% of the original.

In the experiments, all models are trained on the computer with configurations of Intel Xeon Silver 4208 Processor @ 2.10 GHz, 8 cores and 32GB RAM, 24GB NVIDIA GeForce RTX 3090.

### 3.2 Performance comparsion with other methods on benchmark datasets

To evaluate the performance of IGEGRNS, we apply the proposed GRN inference method to six time-series scRNA-seq datasets, including hESC, hHEP, mESC, mHSC-E, mHSC-GM, and mHSC-L. We compared it with nine competing algorithms, which have been proven to achieve good performance. According to the previous comparative analysis, we select GENIE3, GRNBoost2, PIDC, SCODE, and DeepSEM from the existing unsupervised methods. Specifically, GENIE3 and GRNBoost2 both adopt tree-based regressions to determine the gene sets that are coexpressed with TFs. PIDC method is based on multivariate information theory. SCODE applies ordinary differential equations to infer GRN. DeepSEM jointly models the GRN and the transcriptome by generating the SEM with a beta-VAE framework. For the supervised methods, CNNC, GNE, DeepRIG, and DGRNs are included in the comparison. CNNC is a supervised GRN inference method based on deep CNNs. GNE applies multi-layer perception to encode gene expression profiles to predict gene interactions. DeepRIG infers GRNs through prior knowledge generated by WGCN and graph autoencoder GAE. DGRNS is a hybrid deep learning models based on CNNs and recurrent neural networks. Following the BEELINE framework, we consider only highly variable TFs and the top 500 and 1000 most differential genes for each dataset. We take the cell-type-specific network as the ground truth to evaluate the inferred GRNs. The widely used AUROC and AUPRC are adopted as the evaluation metrics.

**Figure 3.**
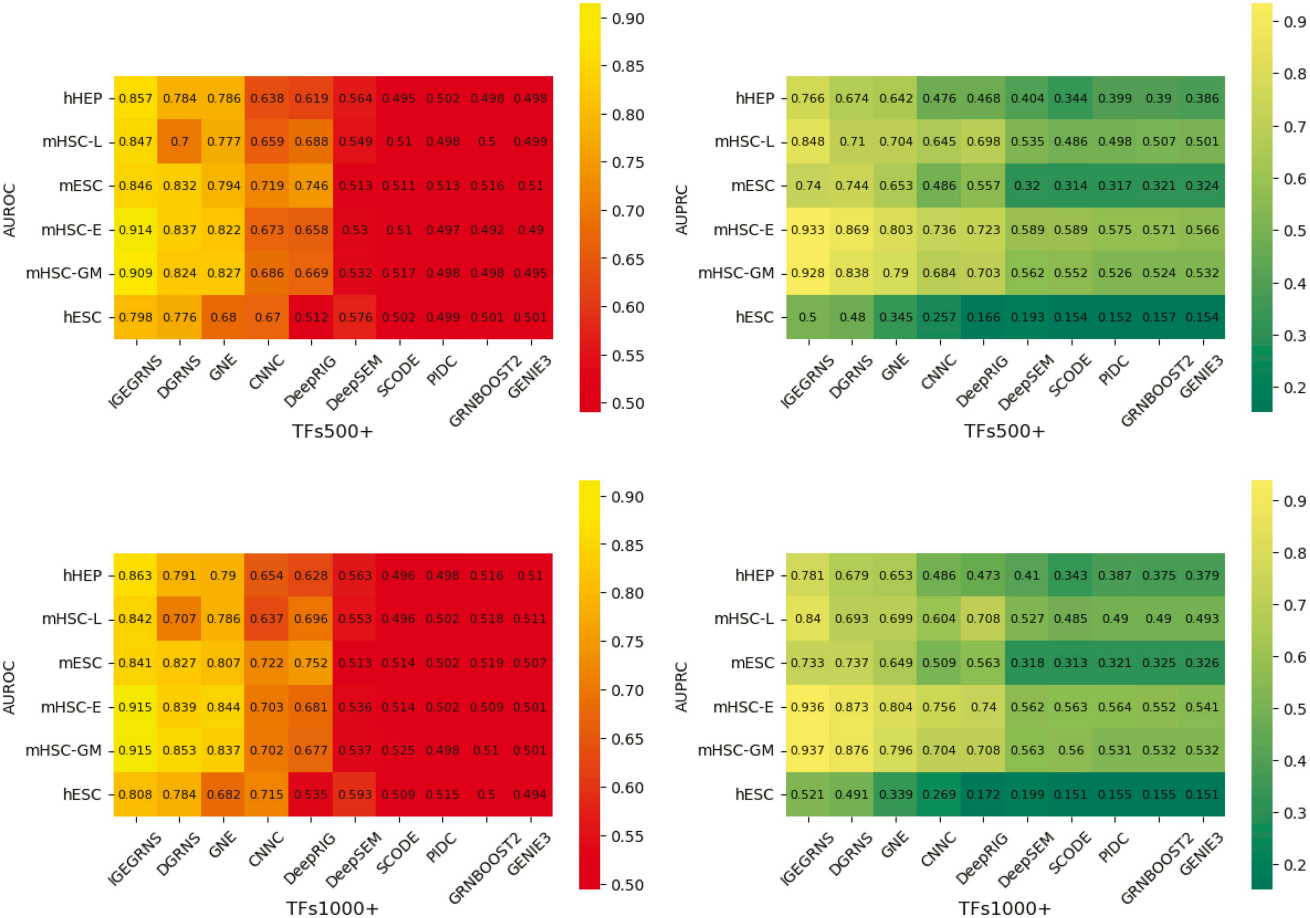
AUROC and AUPRC scores of different GRN inference algorithms across the six scRNA-seq datasets. The left panels display AUROC scores for 500 and 1000 TFs datasets, while the right panels display AUPRC scores for the same datasets. The vertical axis represents the six scRNA-seq datasets, and the horizontal axis represents different GRN inference algorithms

Figure 3 shows the performance of these compared methods on the six scRNA-seq datasets. Overall, the proposed method IGEGRNS achieves the highest AUROC on all these datasets. AUPRC outperforms the compared methods on five datasets except the mESC dataset. In scenarios involving 500 TFs, IGEGRNS demonstrates an average AUROC improvement of 5.6% and an AUPRC improvement of 6.7% compared to the second-ranked algorithm. The highest AUROC improvement 8.2% is achieved on the mHSC-GM dataset, while the highest AUPRC improvement 13.8% is observed on the mHSC-L dataset. In situations with 1000 TFs, our method improves AUROC by 5.0% and AUPRC by 6.6% compared to the suboptimal algorithm. The highest AUROC improvement 7.2% is observed on the hHEP dataset and the highest AUPRC improvement 14.1% is occurred on the mHSC-L dataset. In addition, the proposed method exhibits substantial improvements in both AUROC and AUPRC compared to the unsupervised method, demonstrating the advantage of supervised algorithms over unsupervised ones.

### 3.3 Performance comparsion among different model structures

The results show that IGEGRNS outperforms the compared unsupervised and supervised methods. To verify the reasonability and feasibility of each module of IGEGRNS, we further conduct a comparative analysis to evaluate the effectiveness of different model variants. As shown in Table 1 and Table 2, we compare the performance of IGEGRNS with different aggregation functions, different *k* values for Top-k pooling in the Learning gene node embedding module, and reduce the number of network layers to one simplified CNN in the Predicting gene interactions module. The results demonstrate that the simple MEAN aggregation function is most effective for aggregating the neighboring nodes. Although LSTM is more complex, it might encounter difficulties in extracting effective information of nodes and their neighbors for this task. The max function might miss part information of neighboring nodes. For the stacked CNN, replacing the 3-layer stacked CNN with a simple 1-layer CNN results in an obvious decrease in performance. The results indicate that the simple 1-layer CNN cannot replace the role of the 3-layer stacked CNN, which can extract higher-level features from the complex spatial structure with local features. We also remove the nodes selected by Top-k pooling, and the performance of IGEGRNS without Top-k pooling significantly decreased. This is probably because Top-k pooling not only selects the nodes with the greatest global impact but also serves as a constraint for the model parameters, reducing the possibility of overfitting.

**Table 1.** The AUROC of different model variants on the six scRNA-seq datasets with 1000 TFs.^a^

AUROC of different model structures	hHEP	mHSC-L	mESC	mHSC-E	mHSC-GM	hESC
Aggregator selection=MEAN(IGEGRNS)	0.863	0.842	0.841	0.915	0.915	0.808
Aggregator selection=LSTM	0.853	0.829	0.826	0.904	0.908	0.774
Aggregator selection=MAX	0.854	0.829	0.836	0.903	0.908	0.774
3-layer stacked CNN(IGEGRNS)	0.863	0.842	0.841	0.915	0.915	0.808
1-layer CNN	0.851	0.825	0.829	0.901	0.895	0.801
Top-k pooling, *k* = 1(IGEGRNS)	0.863	0.842	0.841	0.915	0.915	0.808
Without Top-k pooling	0.845	0.837	0.828	0.901	0.909	0.802

a By default, IGEGRNS uses the MEAN aggregator, 3-layer stacked CNN, Top-k pooling with *k* = 1.

**Table 2.** The AUPRC of different model variants on the six scRNA-seq datasets with 1000 TFs.^a^

AUPRC of different model structures	hHEP	mHSC-L	mESC	mHSC-E	mHSC-GM	hESC
Aggregator selection=MEAN(IGEGRNS)	0.781	0.840	0.733	0.936	0.937	0.521
Aggregator selection=LSTM	0.776	0.820	0.693	0.923	0.929	0.469
Aggregator selection=MAX	0.776	0.819	0.711	0.923	0.926	0.469
3-layer stacked CNN(IGEGRNS)	0.781	0.840	0.733	0.936	0.937	0.521
1-layer CNN	0.763	0.813	0.710	0.922	0.909	0.515
Top-k pooling, *k* = 1(IGEGRNS)	0.781	0.840	0.733	0.936	0.937	0.521
Without Top-k pooling	0.748	0.828	0.722	0.921	0.928	0.518

a By default, IGEGRNS uses the MEAN aggregator, 3-layer stacked CNN, Top-k pooling with *k* = 1.

**Figure 4.**
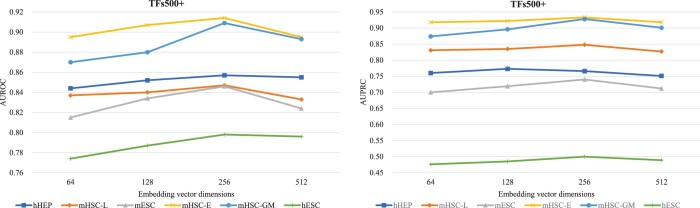
Comparison of AUROC and AUPRC across six scRNA-seq datasets with 500 TFs, varying embedding vector dimensions. The embedding dimensions are respectively set to 64, 128, 256, and 512

To explore how embedding vector dimensionality affects gene interaction prediction, we further conduct a comparative analysis of IGEGRNS performance with four different embedding vector sizes, including 64, 128, 256, and 512. The results are presented in Fig. 4. As the dimensionality increases, the performance of IGEGRNS improves initially, reaches its peak at size 256, and then declines. This trend suggests that smaller embedding vectors may lead to information loss, while larger ones could introduce redundant information and noise, posing challenges for CNNs in extracting key features. Consequently, for optimal performance, we set the embedding vector dimensionality to 256 as the default experimental setting. Furthermore, we compare the performance of aggregating 1-hop, 2-hop, and 3-hop neighboring nodes on these six datasets. The results indicate that aggregating 1-hop neighbors achieves better performance with less computation cost. For example, on the dataset hHEP, the AUROC with 1-hop, 2-hop, 3-hop neighboring nodes are respectively 0.84, 0.77, 0.75. The AUPRC with 1-hop, 2-hop, and 3-hop neighoring nodes are respectively 0.74, 0.67, 0.66.

### 3.4 The analysis of inferred GRNs on real datasets

To further validate our proposed method, we train the model on the training set and infer the GRNs on two real datasets. The first dataset was derived from the direct reprogramming process of mouse embryonic fibroblasts (MEFs) to myofibroblasts. This dataset contains 405 cells, which were measured at 0, 2, 5, and 22 days, respectively (Treutlein *et al.* 2016). The second dataset was derived from the differentiation process of hESCs from qualitative endodermal cells. This dataset was measured at 0, 12, 24, 36, 72, and 96 h, respectively (Chu *et al.* 2016), and it contains 758 cells. Similar to SCODE (Matsumoto *et al.* 2017), we use the TFs in Riken TFDB for mouse (Kanamori *et al.* 2004) and animalTFDB for human (Zhang *et al.* 2015), and select the top 100 genes with large expression differences for each dataset. To validate the accuracy of the inferred GRNs on these datasets, we compare them with GRNs provided in the regulatory database (http://www.regulatorynetworks.org) (Neph *et al.* 2012, Stergachis *et al.* 2014), constructed from DNase footprints and motifs. A total of 666 directed regulatory edges exist in the first reference network and 376 directed regulatory edges exist in the second reference network. During the training and prediction process, we randomly select 20% of real directed regulatory edges as positive samples and equal-sized negative samples as training data. The AUROC of our proposed model on the two datasets are 0.672 and 0.741, while the AUPRC values are 0.447 and 0.452, respectively.

**Figure 5.**
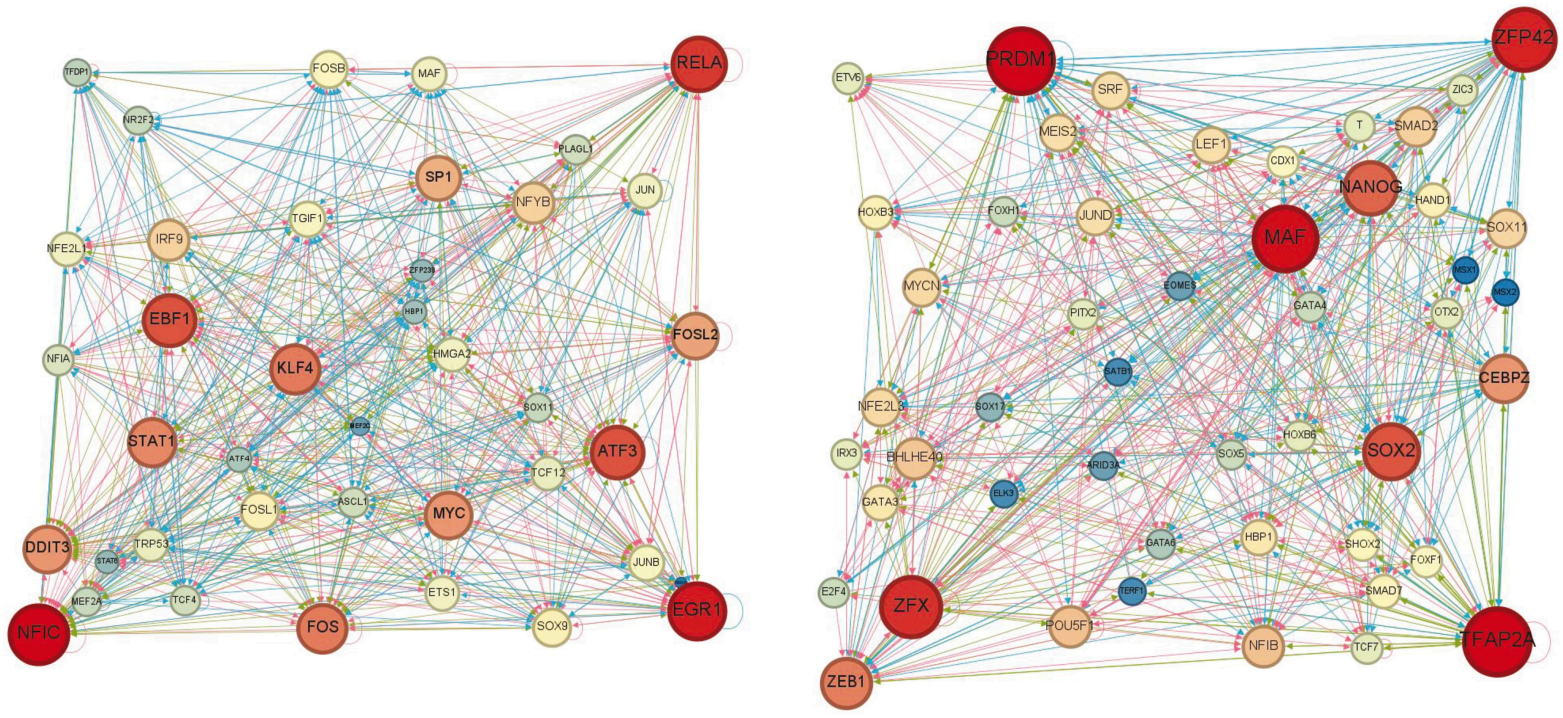
The inferred gene regulatory networks during the process of mouse embryonic fibroblasts (MEF) to myoblasts (the left subfigure) and the process of differentiation of human embryonic stem cells derived from qualitative endodermal cells (the right subfigure). Gene nodes are colored from light to dark in descending order of magnitude. Edges colored in three colors, where red indicates edges that are present in the ground-truth networks and are predicted to be positive by the model, green indicates edges that are present in the ground-truth networks but are predicted to be negative by the model, and blue indicates edges that are not present in the ground-truth networks but are predicted to be negative by this model. According to the inferred GRNs, MYC genes and EBF1 genes play important roles during direct reprogramming of mouse embryonic fibroblasts (MEF) to myofibroblasts. HAND1 and TCF7 genes play important regulatory roles in the gene regulatory network during the differentiation process of human embryonic stem cells from endodermal cells

The visualization results are shown in Fig. 5, where the gene nodes are shaded from light to dark based on degree, and the edges are colored in three colors. Red represents edges that are present in the ground-truth networks and are predicted to be positive by the model, green indicates edges that are present in the ground-truth networks but are predicted to be negative, and blue indicates edges that are not present in the ground-truth networks but are predicted to be negative by the model. For the GRN during direct reprogramming of MEF to myoblasts, there are 244 red edges, 256 green edges, 256 blue edges, and the sparsity of the whole network is 0.31. According to the inferred GRN, we observe that genes MYC and EBF1 played significant regulatory roles, with MYC positively regulating 36 genes and EBF1 regulating 35 genes. MYC has been identified as a regulator that directly or indirectly activates genes associated with muscle cell specificity. It is implicated in regulating cell proliferation rates, thereby facilitating the transformation of embryonic fibroblasts into myoblast and promoting muscle differentiation (Luo *et al.* 2019). Similarly, EBF1 plays a direct role in muscle cell differentiation by promoting the expression of muscle cell-specific genes and modulating signaling pathways pertinent to muscle cell differentiation (Jin *et al.* 2014). For the GRN during the differentiation process of hESCs in qualitative endoderm cells, there are 149 red edges, 226 green edges, 226 blue edges, and the sparsity of the whole network is 0.17. Based on the inferred GRN, we observe that HAND1 and TCF7 are important to the differentiation process of hESCs in qualitative endoderm cells. Specifically, HAND1 functions as a TF governing the directional differentiation of endodermal cells into specific cell lines (Riley *et al.* 1998, Firulli *et al.* 2020). It contributes to maintaining the specificity and function of qualitative endodermal cells through the regulation of specific gene expressions. TCF7 plays a crucial role in embryonic development and cell fate determination. Additionally, TCF7 influences the state and function of embryonic stem cells by regulating the expression of genes associated with stem cell properties (Liang and Liu 2018, Sierra *et al.* 2018).

## 4 Discussion

Due to the complexity and uncertainty of regulatory relationships, it is still a challenge task to infer the regulatory relationships across multiple time nodes. In this paper, we develop a supervised deep learning framework, IGEGRNS, to infer GRNs from scRNA-seq data based on graph embedding. In the framework, contextual information of genes is captured by GraphSAGE, which aggregates gene features and neighborhood structures to generate low-dimensional embedding for genes. Then, the *k* most influential nodes in the whole graph are filtered through Top-k pooling. Finally, potential regulatory relationships between genes are predicted by stacking CNNs. The experimental results demonstrate that IGEGRNS outperformed nine competing methods on six cell-specific scRNA-seq datasets. The proposed IGEGRNS method shows promising results in terms of inferring GRNs and biological interpretability, implying the high-level relation information among genes and the information of the node neighbors are effective in GRN inference. In the future, with the continued advancement of deep learning models, we will further introduce more effective models to learn feature representation from complex data, and accurately predict the regulatory relationships among genes.

## Data Availability

All data used in this study are available in the public database. Gene expression raw data are available in NCBI GEO database under the following accession numbers: human embryonic stem cells (hESC): GSE75748, human mature hepatocytes (hHEP): GSE81252, mouse embryonic stem cells (mESC): GSE98664, mouse blood stem/progenitor cell (mHSC): GSE81682. Reference networks and processed gene expression data are available at https://doi.org/10.5281/zenodo.3378975.
